# A BLE-Based Pedestrian Navigation System for Car Searching in Indoor Parking Garages

**DOI:** 10.3390/s18051442

**Published:** 2018-05-05

**Authors:** Sheng-Shih Wang

**Affiliations:** Department of Information Management, Minghsin University of Science and Technology, Hsinchu 30401, Taiwan; sswang@must.edu.tw

**Keywords:** positioning, navigation, car-searching, Bluetooth Low Energy (BLE)

## Abstract

The continuous global increase in the number of cars has led to an increase in parking issues, particularly with respect to the search for available parking spaces and finding cars. In this paper, we propose a navigation system for car owners to find their cars in indoor parking garages. The proposed system comprises a car-searching mobile app and a positioning-assisting subsystem. The app guides car owners to their cars based on a “turn-by-turn” navigation strategy, and has the ability to correct the user’s heading orientation. The subsystem uses beacon technology for indoor positioning, supporting self-guidance of the car-searching mobile app. This study also designed a local coordinate system to support the identification of the locations of parking spaces and beacon devices. We used Android as the platform to implement the proposed car-searching mobile app, and used Bytereal HiBeacon devices to implement the proposed positioning-assisting subsystem. We also deployed the system in a parking lot in our campus for testing. The experimental results verified that the proposed system not only works well, but also provides the car owner with the correct route guidance information.

## 1. Introduction

Parking management is an important issue in intelligent transportation systems (ITSs), largely due to the growing number of cars worldwide. In parking management, parking guidance in order to facilitate the search for vacant parking spaces is a fundamental service, and prior studies have proposed many options to address this issue [1,2]. Car-searching is also an essential service when car owners attempt to find their parked cars. As the parking layout of parking facilities is often quite uniform in the indoor parking garage, and may consist of hundreds or thousands of parking spaces, it is difficult for car owners to memorize and identify their parking spaces. In addition, car owners with poor sense of direction may lose their ways to the spaces of their parked cars, and therefore may not find the car [3]. Significantly, if not using an efficient navigation strategy, the car owner is likely to come into difficulties when searching for the parked car.

Recently, studies have investigated the issue of pedestrian navigation [4,5,6,7]. Typically, pedestrian navigation focuses on how to plan the guidance of the route to the destination effectively and efficiently. The solution to the navigation of car searching is similar to that of representative pedestrian navigation. For car searching, the following three tasks should be performed: positioning, route planning, and guidance information presentation. Positioning aims to identify the location of the pedestrian and parking space. Route planning involves the determination of the route from the car owner’s location to the parking space of the car. Given the planned route and environmental information (e.g., a map or floor plan), the presentation of guidance information physically shows the planned route and/or direction to the car owner, possibly in visual, audio, haptic, or hybrid forms [4].

The global positioning system (GPS) is a conventional and widely used approach for user localization but may not be accurate for indoor environments because of the indoor shadowing effect [8]. Prior studies have proposed feasible mechanisms for indoor positioning [9,10,11,12]. The mechanisms use information and communication-based technologies, such as infrared [9], Wi-Fi [11], Bluetooth [12], and the vision-based method [10]. The micro-location technique based on beacon technology provides another localization technique in the indoor environment [13,14,15,16]. Beacon technology operates over Bluetooth Low Energy (BLE), which is a wireless form of technology with low power and low cost, and is presently considered as the primary form of wireless technology in mobile devices [17,18]. As a result, beacon technology is regarded as an appropriate indoor positioning solution, and a comprehensive survey of BLE-based indoor positioning mechanisms for smartphones was proposed in [19].

Mobile devices have currently become prevalent for human beings, and thus it is quite suitable for car owners to use a mobile device to obtain navigation information. The majority of the existing approaches utilize the whole map along with the determined route as the output modality of navigation information [20,21,22]. However, the whole map may include unnecessary information for users, therefore increasing their burden when walking toward their destinations. To solve this problem, previous studies have introduced an intuitive navigation scheme known as turn-by-turn navigation [23,24]. The main concept of the scheme is to provide users with the necessary navigation information, such as directions with the distance and time to turn based on the physical world.

The paper proposes a BLE-based pedestrian navigation system, called BLE-PNS, for car searching in indoor parking garages. As car owners search for their own cars and walk toward the spaces where their cars are parked, this paper uses the term “pedestrian” to represent the term “car owner” hereafter. The main concept of BLE-PNS includes self-guiding and effortless navigation. Self-guiding indicates that pedestrians obtain the navigation information according to their mobile devices instead of the facilities of parking garages, e.g., liquid-crystal display (LCD) or light emitting diode (LED) display boards). Effortless navigation implies that the proposed system provides intuitive and accurate navigation information to pedestrians. Recall that the GPS coordinates (i.e., latitude and longitude) are inappropriate for indoor positioning. Thus, in BLE-PNS, we deployed numerous location-aware devices, called anchors, to assist pedestrians to determine their locations, and introduced a local coordinate system to identify the locations of parking spaces and anchors. In addition, BLE-PNS uses the “walk-straight-first” strategy to derive the shortest route with the minimum number of changing directions (i.e., turning left or right) at intersections as the optimal guidance route.

With respect to navigation information provision, the proposed BLE-PNS considers the “turn-by-turn” navigation information rather than the map of the whole parking garage with a thorough guidance route for pedestrians. The turn-by-turn strategy is defined as a navigation scheme which only provides users with the guidance indication of the walking direction at the next intersection. Specifically, in our navigation strategy, if a pedestrian does not approach the parking space of the parked car, the mobile device provides an indication with respect to the walking direction (walk straight, turn right, or turn left) at the next intersection. Otherwise, the mobile device provides the local map and emphasizes the parking space where the pedestrian has parked his/her car. The BLE-PNS also proposes an orientation correction scheme to avoid the misunderstanding of navigation information resulting from the “might-mill-around-and-get-off-course” movement of pedestrians. The scheme provides an instant hint to the pedestrian when the pedestrian’s heading orientation does not conform to the indicated direction derived from the proposed system.

In this study, we considered the parking lot in the campus of Minghsin University of Science and Technology as the testing field and conducted a series of field trials to evaluate the performance of the proposed system. We implemented the prototype of the proposed system, which deploys enough anchors in the predetermined locations of the field and develops the car-searching mobile app running on an Android-based smartphone. The anchor’s location is represented by a pair of the coordinates of the proposed local coordinate system. The anchors use the BLE technology to communicate with the pedestrian’s smartphone. The field test results showed that the proposed BLE-based pedestrian navigation system can correctly identify the location of the pedestrian. Moreover, the pedestrian can not only obtain the correct route guidance information when walking toward the space of the parked car but also receive instant indication when the pedestrian’s heading orientation is incorrect. We also evaluated the performance of the proposed route planning scheme. Simulation results validated that the proposed route planning scheme outperforms the traditional Dijkstra’s algorithm in the number of changing directions.

The rest of this paper is organized as follows. Section 2 formulates the system model and gives an overview of the proposed system. Section 3 elaborates the design and implementation of the proposed BLE-based pedestrian navigation system. Section 4 shows the field testing and simulation results. Section 5 provides concluding remarks.

## 2. Preliminaries

### 2.1. System Model

**Figure 1:**
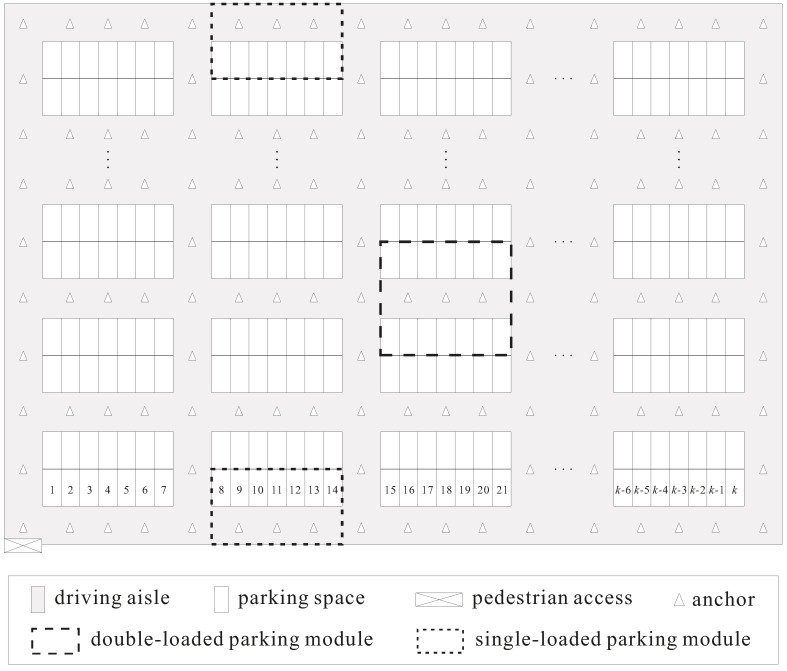
Parking garage layout of the proposed system.

The parking garage considered in this study is a rectangular site and is comprised of the parking area and driving aisles. The parking area consists of many perpendicular parking spaces (i.e., 90° angle parking spaces), one of which is in a single- or double-loaded parking module. The parking modules with parking spaces on one side and both sides of the driving aisle are called single- and double-loaded parking modules, respectively. The numbers of parking spaces in all single-loaded parking modules are identical. Similarly, all double-loaded parking modules have the same number of parking spaces. Assume that the depth of a parking space is twice its width. Then, each parking space has a unique number, and the numbers of parking spaces are sequentially assigned from left to right and bottom to top. All aisles in the parking garages are categorized into vertical and horizontal aisles. The parking garage has only one pedestrian access, which is at the bottom left corner of the parking garage. Assume that the pedestrian enters the parking garage form the pedestrian access and walks alongside the driving aisle when moving toward the parking space of the parked car. We define the target parking space and target parking module as the parking space comprising the pedestrian’s car and the parking module including the target parking space, respectively. Figure 1 illustrates a parking garage layout, in which the number of parking spaces in a single row of a parking module is assumed to be 7.

Recall that this study deployed numerous location-aware anchors in the parking garage for pedestrian positioning. In general, the number of deployed anchors influences the positioning accuracy. The massive deployment of anchors is an intuitive but not efficient method for parking garages with uniform layout. Existing studies have discussed the deployment strategies of positioning-assisting devices [25,26]. Considering that the parking garage used in this study is uniform, we deployed enough anchors by using a strategy in which either the horizontal aisle of a parking module or the vertical aisle of the adjacent parking modules has one or more anchors deployed, respectively, and each intersection of two aisles of the adjacent parking modules also has one anchor. This deployment strategy ensures that the pedestrian at any location of the aisle is able to receive the message sent from the anchors. As shown in Figure 1, either the horizontal aisle of the parking modules or the vertical aisle of the adjacent parking modules has only one anchor.

The anchor deployed in the parking garage periodically broadcasts the location packet including its local coordinates. To receive the location information from anchors, we assume that pedestrians’ smartphones have built-in Bluetooth modules and pedestrians walk toward the target parking space according to the route guidance advised by the proposed car-searching app. This study does not mention issues such as the locations and numbers of garage entrances and exits, driving aisle type (i.e., one- or two-way), and the travel direction of driving aisles, because the focus is only on the moving behavior of pedestrians.

### 2.2. System Overview

The proposed BLE-PNS consists of positioning-assisting and car-searching subsystems. The positioning-assisting subsystem provides the car-searching subsystem with the locations of anchors via location packet dissemination. The car-searching subsystem is the mobile app, which deals with the tasks of local coordinate mapping and route guidance planning according to the information provided by the positioning-assisting subsystem. In addition, the car-searching subsystem takes charge of target parking space setup, user orientation correction, and guidance information presentation. The positioning-assisting and car-searching subsystems use the BLE technology to transmit and receive the location packets, respectively.

**Figure 2:**
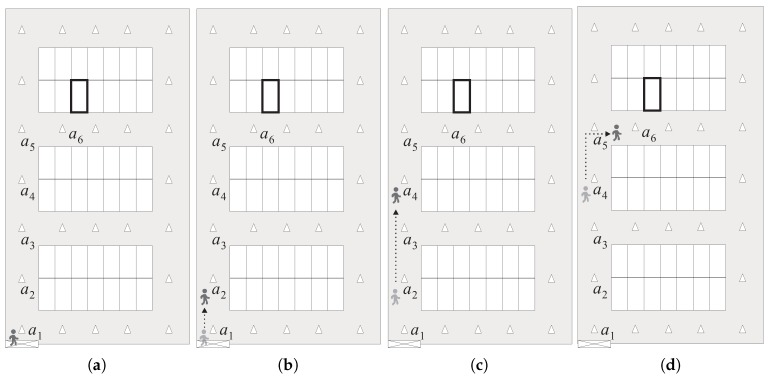
Overview of the proposed navigation system. (**a**) The pedestrian enters the parking garage (i.e., the pedestrian is located around the pedestrian access); (**b**) The pedestrian is located in the aisle section where anchor $a_{2}$ is deployed; (**c**) The pedestrian is located in the aisle section where anchor $a_{4}$ is deployed; (**d**) The pedestrian approaches the target parking module (i.e., the pedestrian enters the aisle belonging to the target parking module).

Figure 2 illustrates a brief overview of the proposed BLE-PNS. For ease of explanation, we assume that the parking area comprises four parking modules (i.e, two single-loaded and two double-loaded parking modules), and three anchors are evenly deployed at the predetermined locations of the aisle of each parking module. The target parking space is represented by the bold rectangle. Before leaving the parked car, the pedestrian uses the app to record the number of the target parking space. When a pedestrian comes back to the pedestrian access and intends to find the parked car, he/she enters the number of the target parking space. The app then prepares to receive the location packet and detects the pedestrian’s heading orientation. As shown in Figure 2a, when the pedestrian’s smartphone receives the location packet broadcast by anchor $a_{1}$ which is the nearest anchor to the pedestrian access, the app determines the route information from the current location to the target parking space according to the proposed walk-straight-first strategy. Then, the app shows an indication of walking straight at the intersection around the pedestrian access.

As shown in Figure 2b, when the pedestrian enters the vertical aisle in which anchor $a_{2}$ is deployed, his/her smartphone receives the location packet broadcast by either anchor $a_{2}$ or $a_{3}$, and the app shows an indication of walking straight at the next intersection. Then, when the pedestrian’s smartphone receives the location packet broadcast by either anchor $a_{4}$ or $a_{5}$, the app derives that the pedestrian should turn right at the next intersection and shows such an indication, as illustrated in Figure 2c. As shown in Figure 2d, when the smartphone of the pedestrian receives the location packet broadcast by anchor $a_{6}$, the app knows that the pedestrian has already entered the target parking module. Therefore, the app shows the local map of the target parking module and emphasizes the target parking space instead of indicating the moving direction at the next intersection. Moreover, if the pedestrian’s heading orientation does not conform to the system guidance direction, the app will suggest that the pedestrian adjusts his/her heading orientation back to the system guidance direction.

## 3. Proposed BLE-Based Pedestrian Navigation System

In this section, we first present the design issues, including pedestrian positioning, route planning, and guidance information representation in detail, and then describe the implementation issues, such as the function block, software development, and hardware components of the proposed BLE-PNS.

### 3.1. Local Coordinate System

**Figure 3:**
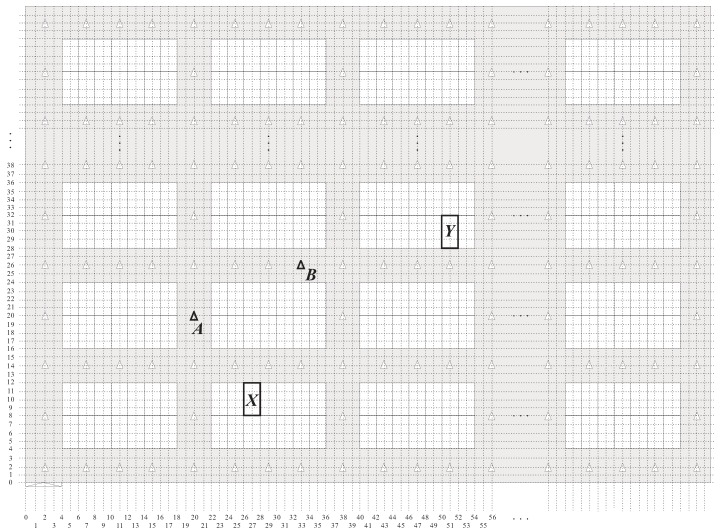
Concept of the proposed local coordinate system.

As the use of the GPS in the indoor environment will most probably cause unexpectable positioning errors due to environmental limitations, the proposed BLE-PNS utilizes a two-dimensional coordinate system called the local coordinate system (LCS) to identify the locations of anchors and parking spaces. In the proposed LCS, the origin point is the lowest leftmost point, and the units of the horizontal and vertical coordinates are assumed to be identical. The width and depth of a parking space are respectively twice and quadruple that of the coordinate unit of the LCS, respectively. Furthermore, the width of an aisle is twice that of the coordinate unit of the LCS. Each parking space or anchor possesses a pair of numbers, that is, the vertical and horizontal coordinates. Figure 3 shows the result of using the proposed LCS to the parking space layout in Figure 1. The locations of the pedestrian access, anchor *A*, anchor *B*, parking space *X*, and parking space *Y* are (2,0), (20,20), (33,26), (27,10), and (51,30), respectively.

In this study, unique local coordinates were assigned to each parking space. However, the proposed LCS is only meaningful for parking-space positioning and not for the pedestrian’s comprehension. That is, pedestrians only know the numbers of parking spaces and not the local coordinates of the parking spaces. Recall that pedestrians provide the target parking space number to the car-searching app when they want to find their parked cars. As a result, the app should be able to translate the parking space number to its corresponding local coordinates by using a one-to-one mapping scheme. The approaches to the representation of the correlation between the parking space numbers and local coordinates are two-fold: static and dynamic mappings. The static mapping scheme stores all pairs of parking space numbers and their corresponding local coordinates in the database. This scheme is simple but requires a considerable amount of storage. The dynamic mapping scheme uses a conversion formula to derive the local coordinates of a parking space according to the parking space number. This scheme is storage-saving and especially suitable for large parking garages because it does not require the storage of the parking space number and local coordinates and only converts the target parking space number to its corresponding local coordinates when necessary.

Let the widths of a parking space and an aisle be denoted as $w_{s}$ and $w_{a}$, respectively. Let $n_{s}$ be the number of a specific parking space, and the local coordinate of this parking space is denoted as $\left(x\left(n_{s}\right),y\left(n_{s}\right)\right)$. Furthermore, let the width of the parking garage be denoted as $W_{g}$, and let $N_{s}^{m}$ indicate the total number of parking spaces on one side of a parking module. Then, we can derive the number of parking modules in a single row ($N_{m}^{row}$) as

(1) $$N_{m}^{row}=\frac{W_{g}-w_{a}}{w_{s}\cdot N_{s}^{m}+w_{a}}.$$

Let $n_{n_{s}}^{row}$ be the serial number of the parking module in a single row containing parking space $n_{s}$ ($n_{n_{s}}^{row}$ = 1, 2, ..., $N_{m}^{row}$ from left to right for each row). Thus, we obtain

(2) $$n_{n_{s}}^{row}=\left\{\begin{array}{cc} \left\lceil \frac{n_{s}}{N_{s}^{m}}\right\rceil \;\mathrm{mod}\;N_{m}^{row} & \mathrm{if}\;\left\lceil \frac{n_{s}}{N_{s}^{m}}\right\rceil \;\mathrm{mod}\;N_{m}^{row}\neq 0\\ N_{m}^{row} & \mathrm{otherwise} \end{array}\right.$$

Let $N_{s}^{row}$ denote the number of parking spaces in a single row. We obtain $N_{s}^{row}=N_{s}^{m}\cdot N_{m}^{row}$. Let $d_{s}$ be the depth of a parking space. By Equations (1) and (2), we can derive $x(n_{s})$ and $y(n_{s})$ as Equations (3) and (4), respectively.

(3) $$x\left(n_{s}\right)=\left\{\begin{array}{cc} w_{s}\cdot \left(n_{s}\;\mathrm{mod}\;N_{s}^{row}\right)+w_{a}\cdot n_{n_{s}}^{row}-\frac{w_{s}}{2} & \mathrm{if}\;n_{s}\;\mathrm{mod}\;N_{s}^{row}\neq 0\\ w_{s}\cdot N_{s}^{row}+w_{a}\cdot n_{n_{s}}^{row}-\frac{w_{s}}{2} & \mathrm{otherwise} \end{array}\right.$$

(4) $$y\left(n_{s}\right)=\left\{\begin{array}{cc} \left(\frac{d_{s}}{2}+w_{a}\right)\cdot \left\lceil \frac{n_{s}}{N_{s}^{row}}\right\rceil & \mathrm{if}\;\left\lceil \frac{n_{s}}{N_{s}^{row}}\right\rceil \;\mathrm{mod}\;2\neq 0\\ \left(\frac{d_{s}}{2}+w_{a}\right)\cdot \left\lceil \frac{n_{s}}{N_{s}^{row}}\right\rceil -2 & \mathrm{otherwise} \end{array}\right.$$

### 3.2. Pedestrian Positioning

To reduce the stress on a pedestrian to reach the target parking space, this study aimed to provide the pedestrian with intuitive but necessary guidance information (e.g., walking direction) instead of complicated information (e.g., route from the pedestrian’s current position to the target parking space). Thus, the car-searching app in BLE-PNS does not need to diagnose the exact position of the pedestrian but only requires knowledge about which aisle section the pedestrian is located in. Recall that in this study, anchors were used to help the car-searching app to determine the pedestrian’s position. In BLE-PNS, we introduced an anchor deployment strategy to ensure that the whole aisle sections can be covered by the signals from anchors. In the strategy, we evenly deployed several anchors in the horizontal aisle of each parking module, and only one anchor was deployed at the central point of the vertical aisle of the adjacent parking modules. Note that the number of anchors deployed in the horizontal aisle is dependent on the accurate rate of positioning. Namely, deploying more anchors can obtain a higher accurate rate of positioning.

**Figure 4:**
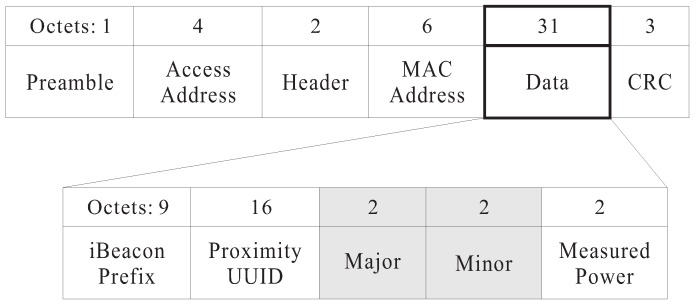
iBeacon advertising packet structure.

In BLE-PNS, each anchor periodically broadcasts a location packet containing its local coordinates to assist in the self-localization of pedestrians’ smartphones. Figure 4 shows the structure of the original iBeacon advertising packet. We modified the contents of the major and minor fields by writing the anchor’s horizontal and vertical coordinates, respectively. According to the Apple iBeacon specification [27], the value of the measured power field indicates the measured signal strength one meter from the sender of the packet; this must be calibrated in advance. If the received signal strength indication (RSSI) equals the measure power value, the estimated distance between the sender and receiver will be 1 m. Let $P_{R}$ be the RSSI measured by a mobile device, and $P_{R}^{1m}$ be the reference RSSI at 1 m (i.e., the value of the measured power field in the iBeacon advertising packet). According to the Android beacon library provided by Radius Networks [28], the estimated distance between the mobile device and a beacon, denoted as $d^{est}$, can be calculated as follows:

(5) $$d^{est}=\alpha \cdot {\left(\frac{P_{R}}{P_{R}^{1m}}\right)}^{\beta }+\gamma ,$$

where α, β, and γ are constants.

To avoid the unnecessary location packets from leading to an incorrect result of pedestrian positioning, the smartphone discards the location packets sent from the anchors far from the pedestrian’s location. Let $d^{th}$ be the maximal distance between a smartphone and an anchor, and $P_{R}^{th}$ be the RSSI corresponding to distance $d^{th}$. By Equation (5), we obtain $P_{R}^{th}={\left(\frac{d^{th}-\gamma }{\alpha }\right)}^{\frac{1}{\beta }}\cdot P_{R}^{1m}$. Therefore, if the RSSI value exceeds $P_{R}^{th}$, the smartphone accepts the packet. Then, the car-searching app retrieves the local coordinates in the received packet and uses them to represent the current position of the pedestrian.

Let $\left(x_{a_{i}},y_{a_{i}}\right)$ be the local coordinates of anchor $a_{i}$ and $P_{R}\left(a_{i}\right)$ be the RSSI value of location frame sent from anchor $a_{i}$. When a smartphone receives the location packet sent from anchor $a_{i}$, the car-searching app estimates its current location, denoted as $\left(x_{a}^{cur},y_{a}^{cur}\right)$ by using Algorithm 1. 

**Algorithm 1:** Pedestrian Position Determination

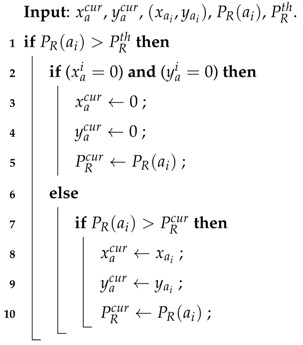



### 3.3. Guidance Information Representation

**Figure 5:**
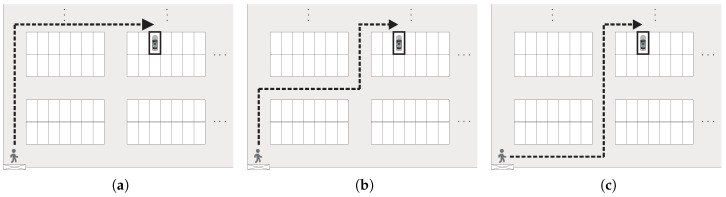
Concept of expected route determination of the proposed system. The target parking space is highlighted by the bold rectangle. (**a**) The route requires one turn to the target parking space; (**b**) The route requires three turns to the target parking space. (**c**) The route requires two turns to the target parking space.

Note that there may be many routes for a pedestrian to reach the target parking space. The proposed BLE-PNS introduces a walk-straight-first strategy to determine an expected route for pedestrians considering the stress and safety of pedestrians during car searching. The walk-straight-first strategy is defined as a route guidance scheme, which considers the walking-straight movement prior to the right/left turn moving unless the user approaches the horizontal aisle of target parking module. This strategy can determine the route with the minimum number of changing directions for users. As illustrated in Figure 5, if a pedestrian needs to move from the pedestrian access to the target parking space without making a detour, three possible shortest routes are available, and measure the same distance. The route shown in Figure 5a requires only one turn, whereas the routes shown in Figure 5b,c require three and two turns, respectively. Therefore, the BLE-PNS will consider the route with one turn as the expected route. In this study, the pedestrian access is located in the bottom left corner of the parking garage; therefore, the target parking space is always located on the right or upper right side of the pedestrian access. According to the aforementioned discussion, pedestrians turn right only once if following the expected route.

Recall that the proposed system aims to provide pedestrians with intuitive and accurate information rather than complicated information to guide them to reach their target parking spaces. The guidance information considered in this study includes the indication of walking direction and orientation correction. Based on the proposed walk-straight-first strategy, we introduced a hybrid approach to indicate walking direction. That is, the proposed system only indicates the walking direction (e.g., walk straight, turn right, or turn left) at the next intersection if the pedestrian has not entered the target aisle. Only when the pedestrian approaches the target parking space does the car-searching app show the local map information of the target parking module with the target parking space emphasized. By using this approach, pedestrians only need to minimally focus on necessary and useful guidance information (i.e., the walking direction at the next intersection), thus being relieved from the stress and confusion caused by unnecessary, complicated information of the entire map and whole routes.

Let $n_{a}^{h}$ be the number of anchors deployed on the horizontal aisle of a parking module, where $N_{s}^{m}\;\mathrm{mod}\;n_{a}^{h}=0$, and $x_{a}^{i}\left(j\right)$ be the horizontal coordinate of the *j*-th anchor (from left to right) in the *i*-th row of parking module. Then, we obtain

(6) $$x_{a}^{j}\left(j\right)=2+\left(N_{s}^{m}\cdot w_{s}+\frac{w_{a}}{2}\right)\cdot \left(i-1\right)+\frac{w_{a}}{2}+\frac{N_{s}^{m}}{n_{a}^{h}}\cdot \left(j-\frac{1}{2}\right)\cdot w_{s},$$

where $j=1,2,\ldots ,\frac{N_{s}^{m}}{n_{a}^{h}}$. Let $n_{a}^{n_{s}}$ denote the serial number of the anchor closest to parking space $n_{s}$ in its parking module. We can derive

(7) $$n_{a}^{n_{s}}=\left\lceil \frac{n_{s}-N_{s}^{m}\cdot \left(n_{n_{s}}^{row}-1\right)}{\frac{N_{s}^{m}}{n_{a}^{h}}}\right\rceil .$$

Let $n_{s}^{tar}$ indicate the number of target parking space. By Equation (7), the serial number of anchor closest to $n_{s}^{tar}$, denoted as $n_{a}^{n_{s}^{tar}}$ can be obtained as

(8) $$n_{a}^{n_{s}^{tar}}=\left\lceil \frac{n_{s}^{tar}-N_{s}^{m}\cdot \left(n_{n_{s}^{tar}}^{row}-1\right)}{\frac{N_{s}^{m}}{n_{a}^{h}}}\right\rceil .$$

As a result, the car-searching app can use Algorithm 2 to determine the correct guidance information that should be shown on the smartphone screen. 

**Algorithm 2:** Guidance Information Determination

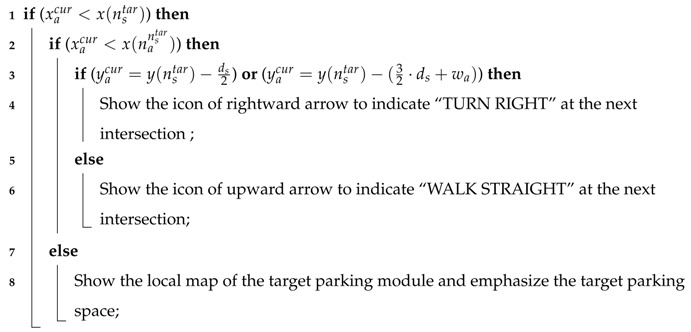



The proposed BLE-PNS includes a function to check whether the pedestrian’s heading orientation conforms to the guidance direction. As this study considered a fixed parking garage, we can calculate the angle (denoted as $\theta_{g}$) between the guidance indication on either the vertical or horizontal aisle and the magnetic north pole in advance. The values of $\theta_{g}$ are 0° and 180° when the guidance indication faces north and south, respectively. In this study, the car-searching app periodically obtained the angle (denoted as $\theta_{h}$) between the pedestrian’s heading orientation and the magnetic north pole through the sensor embedded in the smartphone. Thus, if $|\theta_{h}-\theta_{g}|\geq \varepsilon$, where ε is a pre-determined threshold of the angle, the car-searching app immediately indicates the pedestrian to correct the heading orientation.

### 3.4. System Implementation

**Figure 6:**
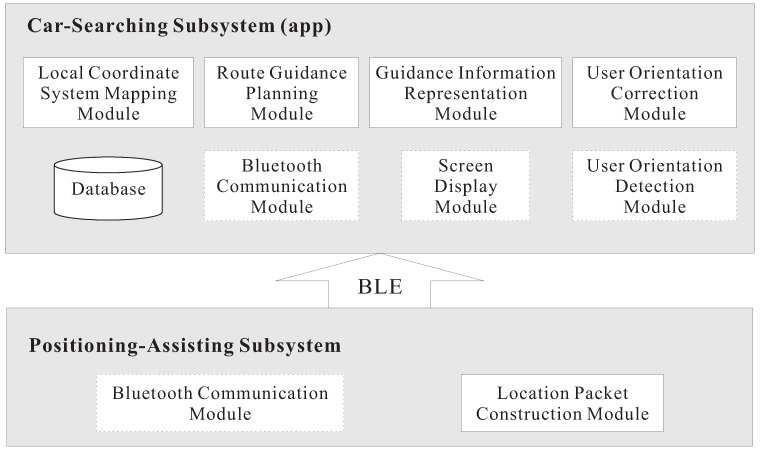
Function blocks of the proposed system. The blocks with solid and dashed lines represent the software and hardware modules, respectively. BLE: Bluetooth Low Energy.

Figure 6 shows the function blocks of the proposed system. The system consists of the positioning-assisting and car-searching subsystems that use the BLE technology to communicate with each other. The car-searching subsystem is an app implemented using Java with Android Studio which is the integrated development environment (IDE) for Google’s Android operating system and installed on an Android smartphone. The mapping module of the LCS transfers the number of parking spaces to the corresponding local coordinates according to Equations (3) and (4). The guidance information presentation module determines the correct indication of either walking direction or local map according to Algorithm 2. To derive $\theta_{g}$ and $\theta_{h}$, the smartphone we used is equipped with the magnetic and accelerometer sensors, included in the orientation detection module. The orientation correction module uses the Android’s getOrientation method to obtain $\theta_{g}$ and $\theta_{h}$ and determines whether the user should change the heading orientation. The indication of either the walking direction or orientation is shown on the screen display module (i.e., smartphone screen). Moreover, we used SQLite as the database in the car-searching subsystem.

The positioning-assisting subsystem uses the Bytereal HiBeacon devices [29] as the anchors. The device uses CR2477 battery for power supply and supports Bluetooth 4.0 (BLE). In addition, it can cooperate with Android 4.3.3 or higher smartphones. The location packet construction module generates the location packet by adding the anchor’s local coordinates into the packet. This can be achieved using the API tool provided by Bytereal when deploying anchors.

## 4. Field Test and Simulation

We considered an outside parking lot at Minghsin University of Science and Technology as the testing field to verify the correctness and effectiveness of the proposed BLE-PNS because there are no appropriate indoor parking garages in the campus for our testing. Although the field is an outside environment, we used the proposed local coordinates rather than the GPS coordinates to identify the locations of anchors and parking spaces.

### 4.1. Experimental Results

**Figure 7:**
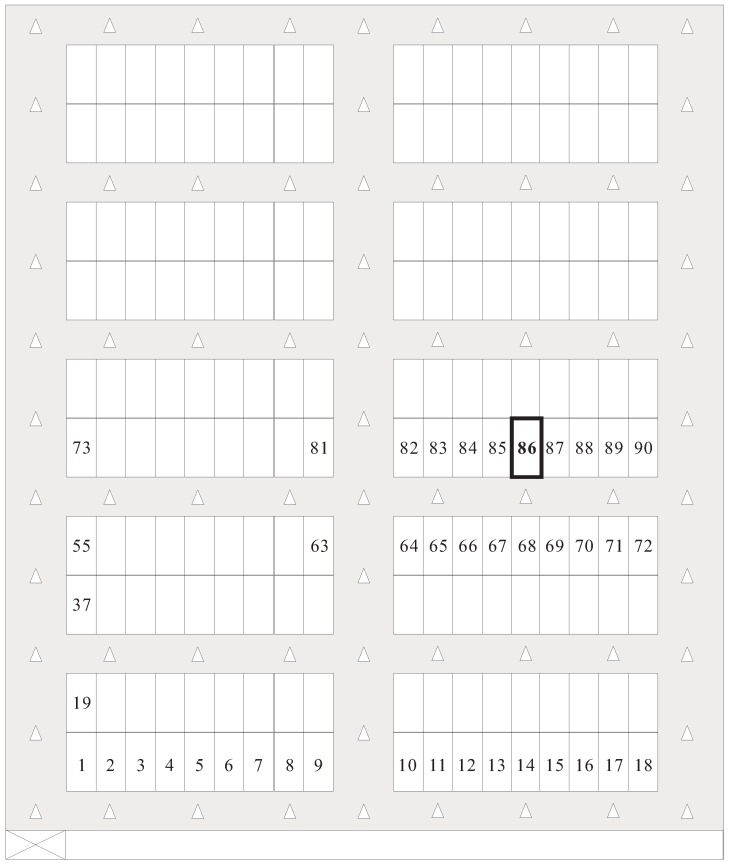
Layout of the testing environment.

Figure 7 shows the layout of the testing field, in which beacon-based anchors are deployed in the horizontal and vertical aisles. We used the Xiaomi Redmi 1S smartphone (Android OS 4.3, Qualcomm Snapdragon 400 MSM8228 processor 1.6 GHz, and 1 GB of RAM) to evaluate our system. Table 1 lists the parameters and their values in the experiments.

**Figure 8:**
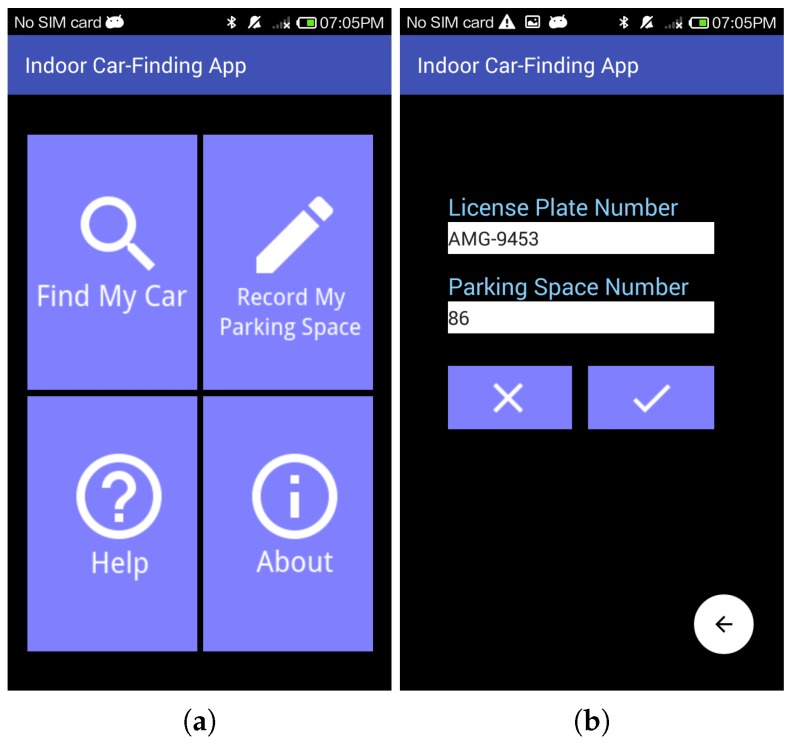
Screenshots of car-searching app. (**a**) Main function interface; (**b**) Interface of input of license plate and parking space numbers.

Figure 8a shows the main screen of the proposed car-searching app. Assume that the car was parked at parking space number 86 (i.e., $n_{s}^{tar}=86$). Before we left the parked car, we activated the car-searching app, pressed the “Record My Parking Space” icon, and entered the license plate and target space numbers. The car-searching app recorded these numbers, as illustrated in Figure 8b. When we returned to the pedestrian access of the parking garage, we activated the car-searching app again and pressed the “Find My Car” icon as shown in Figure 8a, to start the route guidance function. When the “Find My Car” icon was pressed, the app started guiding us turn by turn based on the walk-straight-first strategies according to the location packets received.

**Figure 9:**
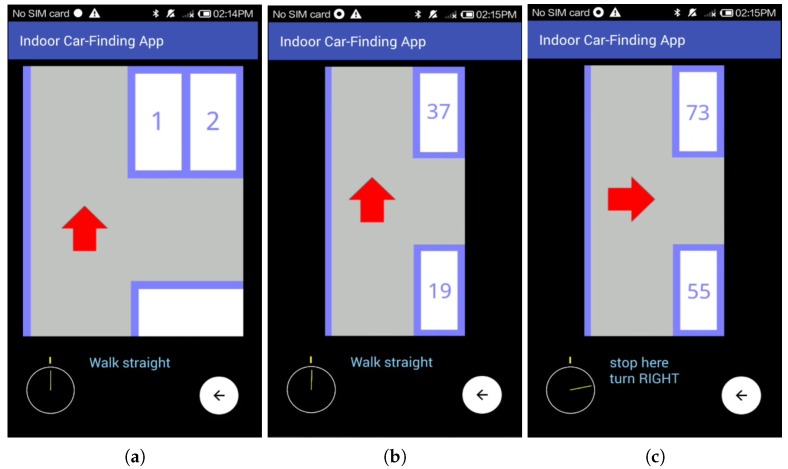
Screenshots of the navigation interface when the pedestrian does not approach the target parking module. (**a**) Shows the upwards red arrow (pedestrian is near the pedestrian access); (**b**) Shows the upwards red arrow (pedestrian is in the vertical aisle around parking space numbers 1 and 19); (**c**) Shows the rightwards red arrow (pedestrian is in the vertical aisle around parking space numbers 37 and 55) and the orientation correction indication.

At first, the car-searching app indicated us to walk straight till the first intersection by showing an icon of a red upward-pointing arrow, as shown in Figure 9a. We then continued walking according to the system indication and entered the vertical aisle. When we were close to the next intersection, the app showed an icon of a red upward-pointing arrow to guide us to walk straight at the next intersection, as shown in Figure 9b. A while later, we noticed the app showing an icon of a red rightward-pointing arrow to hint us to turn right at the next intersection, as shown in Figure 9c. To evaluate the performance of the orientation correction function of the proposed BLE-PNS, at this point, we intentionally turned our heading orientation left more than 10° compared to the system indication. Figure 9c shows that the app detected this situation and indicated us to “stop here, turn RIGHT”.

**Figure 10:**
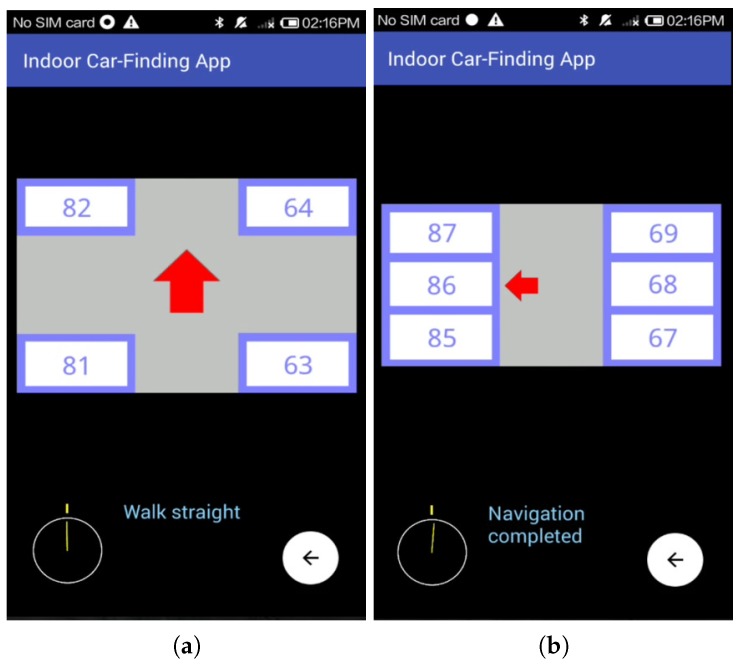
Screenshots of the navigation interface when the pedestrian is close to the target parking module. (**a**) Shows the red upward arrow (pedestrian is in the horizontal aisle of the parking module including parking space numbers 55, 56, …, 63, and 73, 74, …, 81); (**b**) Shows the local map of the target parking module, including six parking spaces, and indicates the target parking space.

When we entered the aisle of the parking module preceding the target parking module, the app still showed a red upward-pointing arrow to advise us to walk straight at the next intersection, as shown in Figure 10a. As shown in Figure 10b, when we entered the aisle of the target parking module, the app showed the map information of this parking module and emphasized the target parking space on the map, and the navigation was successfully completed.

It is noteworthy that before we arrived at the target aisle, the car-searching app only showed a simple indication about the walking direction (walk straight or turn right) at each intersection ahead. Only when we had arrived at the target parking module did the car-searching app present the local map information of the module and emphasize the target parking space. The experimental results show that the proposed BLE-PNS guides us back to the target parking space based on our design, and therefore achieves good performance for correct guidance.

### 4.2. Simulation Results

**Figure 11:**
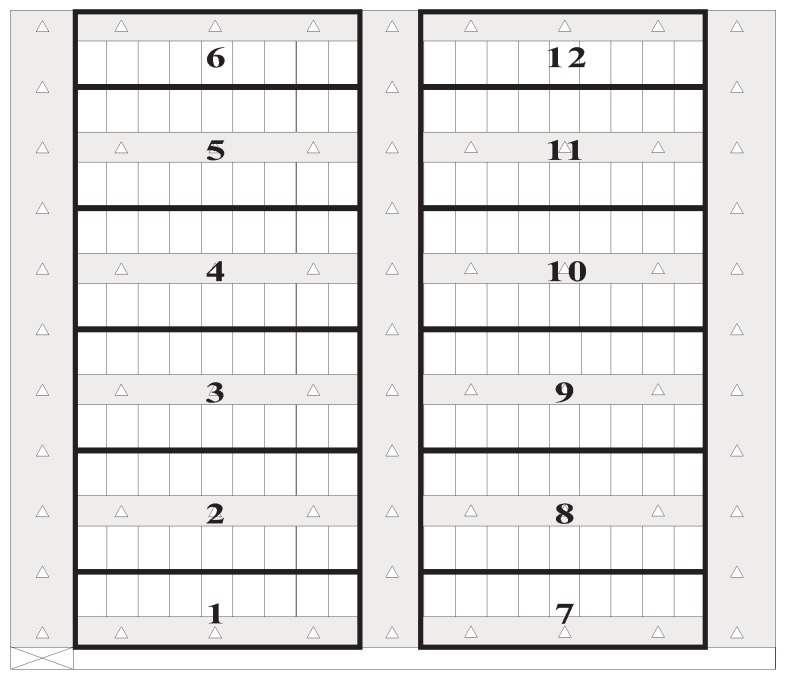
Layout of the simulation environment.

In general, the result of the route planning dominates the performance of the personal navigation system. When not using any navigation approach, the pedestrian will arbitrarily determine the action (walk straight, turn right, or turn left) at the intersection. This strategy is simple, but not an efficient one. Dijkstra’s algorithm is the representative solution to route determination and is used in many applications and previous works. Based on the design concept, we can infer that the proposed turn-by-turn strategy always derives the route with the minimum number of changing directions. In addition to field tests, this study further performs the simulation, considering different target parking modules and using the numbers of changing directions as the metric to evaluate the performance of the proposed route planning scheme and Dijkstra’s algorithm. The simulation environment is shown in Figure 11, which is identical to Figure 7. We consider 12 non-overlapping target parking modules and each parking module is indicated by a unique number (i.e., parking module identifier).

**Figure 12:**
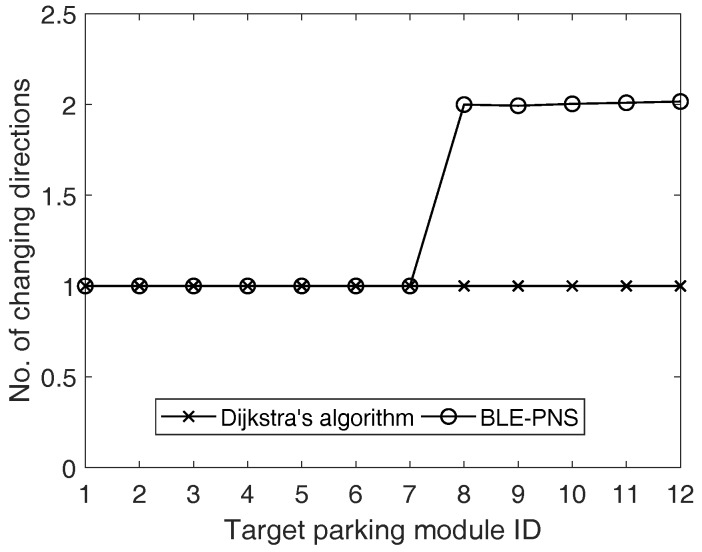
Numbers of changing directions of the proposed system and Dijkstra’s algorithm. BLE-PNS: Bluetooth Low Energy pedestrian navigation system.

The simulation results were averaged over 5000 runs, and results showed that the proposed scheme outperforms Dijkstra’s algorithm because the proposed scheme always determines the route with the minimum number of changing directions, as shown in Figure 12. With the characteristic of scalability, the number of changing directions of the proposed scheme keeps constant. However, the number of changing directions of the Dijkstra’s algorithm increases with the increase of the target parking module identifier.

### 4.3. Discussion

In this section, we discuss the following issues about the design and implementation of indoor pedestrian navigation systems.

#### 4.3.1. Comparisons between BLE-Based and WiFi-Based Positioning Schemes

Compared with the WiFi-based approach, the BLE-based scheme can typically provide higher precision positioning results as the WiFi-based solution probably suffers from significant signal attenuation due to the long distance between the WiFi base station (i.e., access points, APs) and the mobile device. This not only incurs the poor quality of received signals but also reduces the positioning accuracy. In addition, both mobile devices and infrastructures (i.e., beacon-enabled devices) of BLE-based solutions consume less energy compared with the WiFi-based solution, thus extending their operating lifetime. Moreover, deploying beacon-based devices is more flexible than deploying WiFi APs, especially in the indoor environment. The hardware cost (i.e., infrastructure) of BLE-based solution is also lower than that of the WiFi-based approach.

#### 4.3.2. Positioning Accuracy

Recall that unlike most existing solutions, which aim at providing detailed guidance information (e.g., the route from the pedestrian’s current position to the target parking space), the proposed system only provides the pedestrian with the intuitive guidance information (i.e., walking direction) to significantly reduce the stress on pedestrians, especially in the indoor environment. In the proposed system, the car-searching subsystem only requires knowledge about which aisle section the pedestrian is located in instead of the exact position of the pedestrian. Thus, we introduce an anchor deployment strategy to ensure that the whole aisle sections can be covered by the signals from anchors. In the strategy, anchors are evenly deployed in the horizontal aisle of each parking module and only one anchor is deployed at the central point of the vertical aisle of the adjacent parking modules. Because the number of anchors deployed in the horizontal aisle is dependent on the positioning accuracy, more anchors are needed to be deployed if a higher positioning accuracy is required.

#### 4.3.3. Pedestrians’ Walking Speed

The following literature gives the information that the walking speed of humans is at about 1.4 m/s. According to Table 1, the interval of location packets used in the field test is of 100 ms. The interval is much smaller than the value of walking speed of humans. It means that the car owner can actually receive location packets when they are approaching the intersection. Thus, once receiving location packets, the car-searching app installed on the car owner’s smartphone can immediately determine the aisle where the car owner is located according to Algorithm 1. The app then provides the correct guidance information on the screen of the smartphone according to Algorithm 2. Consequently, we summarize that the walking speed is irrelevant to the operation and performance of the proposed system.

## 5. Conclusions

This paper proposed a BLE-based pedestrian navigation system for car searching in indoor parking garages. The proposed system uses an local coordinate system to assist in the localization of parking spaces and anchors. In addition, the system adopts the walk-straight-first and turn-by-turn navigation strategies to guide the pedestrian toward the target parking space. The walk-straight-first strategy derives the shortest route with the minimum number of changing directions at intersections, while the turn-by-turn strategy provides the pedestrian with non-map directions at each intersection before reaching the target parking module. Only when the pedestrian arrives at the aisle of the target parking module does the car-searching app show the local map information of the target parking module and emphasize the target parking space. In addition, the proposed system has the capability to correct the pedestrian’s heading orientation when this orientation does not conform to the system indication. We implemented the prototype of the proposed system and evaluated the system performance. The experimental results verified the correctness and effectiveness of the proposed system. In addition, the simulation results showed that the proposed route planning scheme can determine the route with the minimum number of changing directions compared to the traditional Dijkstra’s algorithm.

## Figures and Tables

**Table 1:** Experimental parameters and values.

Parameter	Value
$w_{s}$	2
$w_{a}$	4
$N_{s}^{m}$	9
$n_{a}^{h}$	3
Output power level of anchors	4 (4 dBm)
Interval of location packets	100 ms
$P_{R}^{1m}$	−59 dBm
$P_{R}^{th}$	−80 dBm
α	0.89976
β	7.7095
γ	0.111
ε	10
